# Identification of Novel Reference Genes Suitable for qRT-PCR Normalization with Respect to the Zebrafish Developmental Stage

**DOI:** 10.1371/journal.pone.0149277

**Published:** 2016-02-18

**Authors:** Yu Hu, Shuying Xie, Jihua Yao

**Affiliations:** State Key Laboratory of Genetic Engineering, Institute of Genetics, School of Life Sciences, Fudan University, Shanghai, China; Oregon State University, UNITED STATES

## Abstract

Reference genes used in normalizing qRT-PCR data are critical for the accuracy of gene expression analysis. However, many traditional reference genes used in zebrafish early development are not appropriate because of their variable expression levels during embryogenesis. In the present study, we used our previous RNA-Seq dataset to identify novel reference genes suitable for gene expression analysis during zebrafish early developmental stages. We first selected 197 most stably expressed genes from an RNA-Seq dataset (29,291 genes in total), according to the ratio of their maximum to minimum RPKM values. Among the 197 genes, 4 genes with moderate expression levels and the least variation throughout 9 developmental stages were identified as candidate reference genes. Using four independent statistical algorithms (delta-CT, geNorm, BestKeeper and NormFinder), the stability of qRT-PCR expression of these candidates was then evaluated and compared to that of *actb1* and *actb2*, two commonly used zebrafish reference genes. Stability rankings showed that two genes, namely *mobk13 (mob4)* and *lsm12b*, were more stable than *actb1* and *actb2* in most cases. To further test the suitability of *mobk13* and *lsm12b* as novel reference genes, they were used to normalize three well-studied target genes. The results showed that *mobk13* and *lsm12b* were more suitable than *actb1* and *actb2* with respect to zebrafish early development. We recommend *mobk13* and *lsm12b* as new optimal reference genes for zebrafish qRT-PCR analysis during embryogenesis and early larval stages.

## Introduction

Quantitative real-time polymerase chain reaction (qRT-PCR) has been widely employed for gene expression analysis because of its specificity, sensitivity and reproducibility [1, 2]. The accuracy of qRT-PCR results depends greatly on the reference genes used and use of appropriate genes could reduce systematic and random errors arising from the amount of the original sample, RNA quality and reverse transcription efficiency [3, 4]. Therefore, the selection of internal reference genes is important for accurate normalization results. Theoretically, an ideal reference gene should maintain a stable mRNA expression level and not change between different developmental stages or due to experimental conditions [5, 6]. However, no single reference gene has been shown to have a universal and constant level. Although some “housekeeping” genes are frequently applied to normalize gene expression [7, 8], many studies have reported that the transcript quantity of these reference genes can vary considerably under different conditions [9–13].

Zebrafish is an excellent vertebrate animal model for molecular genetic studies of development and gene functions [14, 15]. To obtain precise results from qRT-PCR assays related to zebrafish development, a reliable normalization reference gene should be used that is expressed stably with minimal variation in expression levels. However, the commonly used zebrafish reference genes are mainly orthologues of genes stably expressed in mammal tissues, or are identified by systematic comparisons with traditional reference genes [16, 17], and their reliability under certain conditions may be questionable. Recently, on re-assessment of reference gene stability, most of the commonly used reference genes were found to be unsuited for qRT-PCR normalization during zebrafish embryogenesis, even the widely-used housekeeping genes such as *beta-actin* (*actb1)* and *glyceraldehyde-3-phosphate dehydrogenase (gapdh)*, as they were reported to show high variability during different developmental stages [7]. Moreover, for zebrafish development, even those that were thought to be ideal as reference genes, such as *beta-actin2* (*actb2*), also have some drawbacks. As is well known, *actb2* belongs to a large gene family and shares 89% homology with *actb1* over 64% of their length [12], making it difficult to design specific primers for qRT-PCR [18]. Furthermore, the potential existence of pseudogenes closely related to *actb2* also weakens the validity of its use to normalize target datasets [19]. Therefore, it is imperative to identify novel reference genes optimal for zebrafish qRT-PCR analysis during early development.

Deep RNA sequencing (RNA-seq) analysis has become a powerful tool in high-throughput transcriptomic studies with high resolution [20], sensitivity [21], accuracy [22], a low background signal [23] and a large assembly of datasets [24–26]. Recently, RNA-seq datasets have been used directly to explore novel reference genes for model systems and non-model organisms. For example, the *eukaryotic translation elongation factor 1 alpha* (*eEF1A1*) and *inkel-Biskis-Reilly murine sarcoma virus (FBR-MuSV) ubiquitously expressed (FAU*) genes have been newly developed as ideal reference genes for human lung squamous-cell carcinoma [27]. Some suitable reference genes for plants have also been identified, such as *AvrRpt2-induced gene* (*VvAIG1*) and *T-complex 1 beta-like protein* (*VvTCPB*) for *Vitisvinifera* [10], *Postsynaptic protein-related* (*PPR*) and *Guanosine nucleotide diphosphate dissociation inhibitor 1* (*GDI1*) for *Brassica napus*L [18], *AvrRpt2-induced gene* (*gyrA*) and *Translation elongation factor aEF-2* (*fusA*) for *Corynebacterium pseudotuberculosis* [28], *SAND family* (*SAND*) and *N2227-like family protein* (*N2227*) for *Catharanthusroseus* [29] and SL_REF2 and SL_REF5 for the two sexes of *Silenelatifolia* [30]. More recently, several expressed repetitive elements (ERE), such as hatn10, dna15ta1 and loopern4, have been proposed as zebrafish reference targets [31].

In this study, we used our previous RNA-Seq datasets on zebrafish embryos and early larvae [32] to identify the most stably expressed genes during zebrafish early development. We identified four genes with the least amounts of variation in expression levels at 9 developmental stages. These were then considered as novel reference gene candidates. The qRT-PCR expression stabilities of the candidates were further evaluated using four statistical algorithms, namely delta-CT [33], geNorm [34], BestKeeper [35] and NormFinder [36], and compared to those of the commonly used zebrafish reference genes *actb1* and *actb2*. According to their stability ranking, two of the candidates were identified as novel optimal reference genes. Finally, we used the newly selected reference genes to normalize three well-studied target genes during zebrafish embryogenesis, thereby demonstrating their effective use.

## Materials and Methods

### Zebrafish embryos and larvae

Zebrafish (AB strain) used in the experiments were raised and maintained under standard laboratory conditions of 14 h light/10 h darkness at 28.5°C, as described by Westerfield [37]. The stage of the embryos was determined by morphological features according to Kimmel [38]. Nine stages of development of zebrafish embryos and larvae covering seven different periods were used in this study: cleavage (64/128-cell), blastula stage (oblong-sphere), gastrula stage (50%-epiboly), segmentation stage (15-somite), pharyngula stage (36 hpf), hatching stage (48 hpf, 60 hpf, 72 hpf) and early larval stage (1-week) as described previously [32]. The protocol was approved by Shanghai Research Center for Model Organisms (SRCMO-IACUC No: 2009–0001).

### Quantitative real-time polymerase chain reaction (qRT-PCR)

The RNA samples used in the study were the same as those used in our previous RNA-seq. The total RNA was extracted from the afore mentioned stages of (about 2000) embryos or larvae using Trizol (Invitrogen, Carlsbad, CA, USA) methods according to the manufacturer’s instruction. RNA quality was evaluated by gel electrophoresis, with the concentration measured with NanoDrop 2000 (Thermo Scientific Waltham, MA, USA). The aliquots were stored at -80°C [32].

The primers for this study were designed using the primer analysis software Primer3 (http://frodo.wi.mit.edu/primer3/). Reverse transcription was carried out with M-MLV Reverse Transcriptase (Promega, Cat.No.M1705, Madison, WI, USA) using oligo (dT)_15_ primer and random primer. Real-time PCR was performed with EvaGreen dye (Biotium, Cat. No. 31000, Hayward, CA, USA) on an MJ DNA Engine Opticon^™^ System (PTC-200 DNA Engine^™^ Cycler and CFD-3200 Opticon^™^ Detector, New York, NY, USA). When the primers efficiency was tested, the mixture cDNA from several developmental stages were used. For the test PCRs, a twofold serial dilution of the mixture cDNA sample was used and a total of 5 dilutions (1, 2, 4, 8 and 16) were assessed. Those primers with good relationship between input target cDNA concentration and CT (R^2^> 0.99) and with ideal range for slope of qPCR standard curve, as well as with single peak in melt curve analysis were selected. The primers used in this study are listed in S1 Table. The location of the primers and the length of PCR product are as following: the *ssr2* primers pair laid across the exon 4 and exon 6 with the length of PCR product 197bp; the *C1H16orf72* laid across the exon4 with 275bp; the *lsm12b* laid across exon 3 and exon 5 with 299bp; the *mobk13* (*mob4*) laid across exon8 with 297bp; the *actb1* laid across exon 5 and exon 6 with 323bp; the *actb2* laid across exon 5 and exon 6 with 321bp. For the target genes, the *fzd7a* primers pair laid across exon1 with the length of PCR product 359bp; the *sox7* laid across exon 4 with 196bp, and the *szl* laid across exon 3 and exon 4 with 322bp. When in the comparison, the single cDNA sample (from 2000 embryos) for each development timepoint was used. PCR amplification was performed using cDNA as the template and the conditions were 94°C for 3min, 40 cycles of 94°C 15s, 60°C 15s, and 72°C 20 s, 1 cycle of 20°C 2 min. All the qPCR reactions were performed with three parallel samples; the negative control contains no sample template. We analyzed the qPCR data with the Opticon software and analyzed the RNA interfere result using the comparative CT method, utilizing the assumption that the primer efficiencies are relatively similar.

### Statistical analysis

The RPKM values of all 29,291 genes were put into R language (3.1.2) and R-Studio (0.98.1091), and the expressed threshold was set as RPKM = 0.17 [32]. Coefficient of variance [ratios of standard deviation (SD) to the mean (μ)] and normalized RPKM values of selected genes, and Pearson’s correlation coefficient (r) between RNA-seq and qRT-PCR datasets were also calculated by means of R language. In addition, all boxplots, heatmaps, histograms and line graphs were drawn using R. Transcript information of genes was based on Zebrafish Ensembl Release 80 (May 2015, http://asia.ensembl.org/Danio_rerio/Info/WhatsNew?db=core#change_1653).

The qRT-PCR stabilities of these candidate reference genes were evaluated with four stability analysis software programs: delta-CT [33], geNorm [34], BestKeeper [35] and NormFinder [36]. All four software programs were used according to the manufacturer’s instructions.

### Whole-mount in situ hybridization

To study the expression patterns of the four zebrafish candidate reference genes during embryogenesis, a whole-mount in situ hybridization procedure was carried out as described by Westerfield [37]. To facilitate visualization of RNA during in situ hybridization, 0.003% phenylthiourea (PTU) (Sigma) was added to the embryos before 24hours post-fertilization (hpf) to block pigment formation [39]. Using the primers showed in S1 Table, PCR amplification was performed with single-strand cDNA as the template, which was the reverse transcript from oblong/sphere stage embryonic mRNA. The amplified product was then cloned into the pGEM-T Easy vector (Promega). Using T7 and SP6 RNA polymerases, the sense and antisense RNA probes were synthesized and each was labeled with digoxigenin (Roche). Digital images of all embryos were captured using a Zeiss Axio Imager M1 microscope.

## Results and Discussion

### Screening of potential reference genes from zebrafish deep RNA-seq data of embryonic and early larval stages

Searches for reference genes in zebrafish are generally based on identification of orthologues of genes stably expressed in mammalian tissue, mainly from mice or humans [12], such as *β-2-microglobulin* [13], *gapdh* [16] and *β-actin* [17]. However, validation of the expression stability of those genes showed that they may not be applicable for normalization because of their variability at different stages of zebrafish development [9, 11]. In the present study, we screened novel reference genes from our previous RNA-seq dataset (29,291 genes in total) by setting up a series of conditions [32]. First, to obtain genes which are expressed stably in zebrafish during early development, the ratio of the maximum to the minimum RPKM (RPKM_max/min_) at 9 defined stages was kept to less than 2 (< 2), and the coefficient of variation (CV) value was less than 0.3 (< 0.3, p< 0.05). From results of the preliminary screening, 197 genes with a stable expression level were selected (S2 Table).

**Fig 1:**
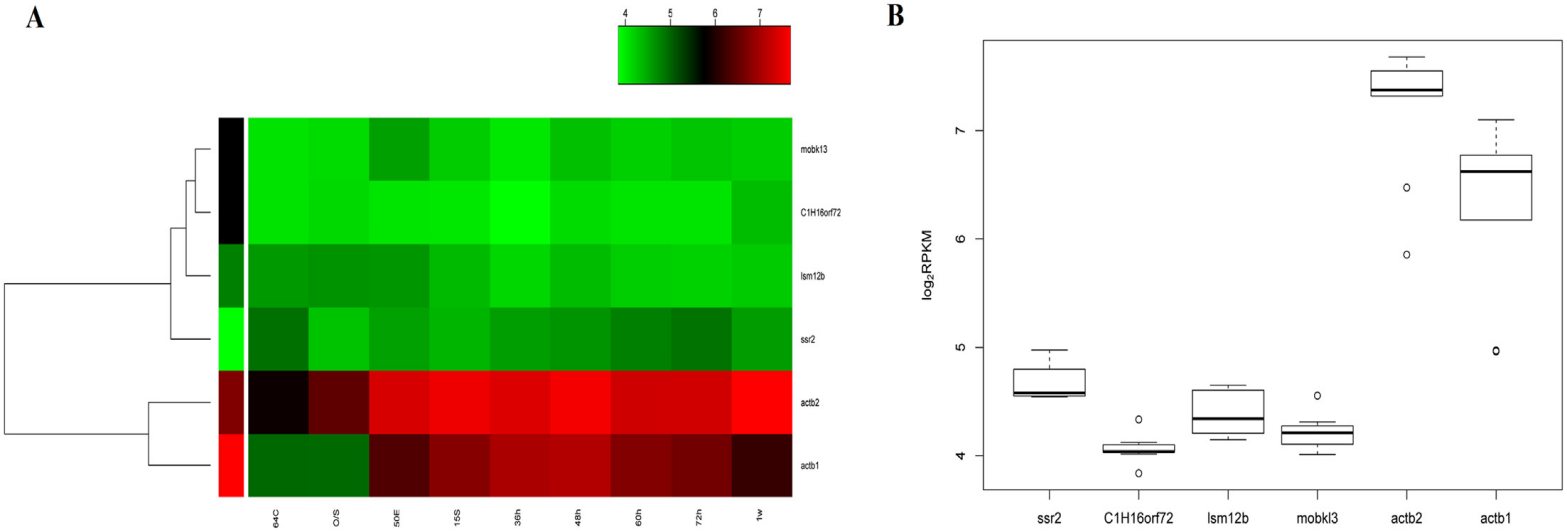
Characteristic expression of the tested genes. (A) A heatmap was used to visualize the expression pattern of the six tested genes at nine stages in zebrafish development. (B) Expression levels and variations of the tested genes at the 9 stages. As shown in four candidate genes, namely *ssr2*, *C1H16orf72*, *lsm12b* and *mobk13*, were more stable than the commonly used reference genes *actb1* and *actb2* during early development.

Since ideal reference genes for zebrafish developmental studies should be moderately or highly expressed at different developmental stages, the minimum RPKM value was set to 40(>40) and it acted as a major parameter for the next round of screening. Twelve genes of the 197 met this requirement (S3 Table). For further validation of expression stability, the top five out of the 12 candidate genes with a minimal RPKM_max/min_ were selected. To facilitate the primer design of each reference gene and ensure the accuracy and reliability of the qRT-PCR results, genes with one transcript, or those with two transcripts that overlapped were retained, while in the case of those with multiple splice isoforms, only one was chosen [40]. The 4 retained candidate genes were *signal sequence receptor beta* (*ssr2*), *C1H16orf72* (zgc:77849), *Like-Sm protein 12 homolog b* (*lsm12b*) and *MOB family member 4*, *phocein* (*mob4*/*mobk13*) as marked with an asterisk (*) in S3 Table. The characterization of the genes is displayed in Table 1. For subsequent tests, the commonly used zebrafish reference genes *actb1* and *actb2* served as the controls. From Table 1, it is evident that the RPKM_max/min_ values of the four candidates (ranging from 1.50 to 1.75), were significantly less than those of *actb1* (8.48) and *actb2* (6.20), indicating that the 4 candidate genes had a more stable expression than that of *actb1* and *actb2* during zebrafish early development, and thus, they may be more suitable as reference genes. According to previous reports, these four candidates may have important functions as shown in Table 1[41–45], although the details remain to be clarified. Fig 1 shows the expression profiles (Fig 1A) and the variations of the six tested genes at the 9 developmental stages (Fig 1B). As seen in the figure, the four candidates were expressed more stably, and the variation in respective expression levels was less than that of both *actb1* and *actb2* during zebrafish embryo development, indicating that these four genes may be more suitable for normalization than actb1 and actb2.

**Table 1:** Characterization of the six tested genes.

Ensembl gene ID	Gene name	CV values	RPKM max/min	Spliced variants	Function
ENSDARG00000005230	*ssr2*	0.17	1.540	3	translocon-associated protein β, TRAP[b], for protein translocation across the endoplasmic reticulum membrane[41]
ENSDARG00000012458	*C1H16orf72*	0.13	1.643	2	Unclear
ENSDARG00000045940	*lsm12b*	0.21	1.655	1	stress granule components[42]; mRNA degradation or tRNA splicing[43]
ENSDARG00000056085	*mobk13*	0.17	1.722	1	spindle focusing[44]; organization of microtubule networks[45]
ENSDARG00000037746	*actb1*	0.56	8.475	6	cytoskeletal protein
ENSDARG00000037870	*actb2*	0.41	6.196	7	cytoskeletal protein

In addition, another factor to consider was the number of potential pseudogenes of the candidates, which may influence the accuracy of the qRT-PCR results [19]. With the BLAST-Like Alignment Tool (BLAT) method used in Ensembl, the number of possible pseudogenes closely related to the tested candidates was calculated, as shown in S4 Table. The table shows that all four genes had far fewer potential pseudogenes than did *actb1* and *actb2*, providing convincing evidence that the candidates were more suitable than both *actb1* and *actb2* for zebrafish gene expression analysis.

### Evaluation of the stability of potential reference genes with four statistical methods

The expression stability of reference genes on qRT-PCR is often evaluated by statistical analysis. Finding the best analytical tool could minimize variations in the original CT data and significantly simplify the selection process of the most stable reference genes [30]. To obtain more reliable results on PCR, both oligo (dT)_15_ primer (OP) and random primer (RP) of the six tested genes were used for qRT-PCR analysis. The primers used in this study are listed in S1 Table.

**Fig 2:**
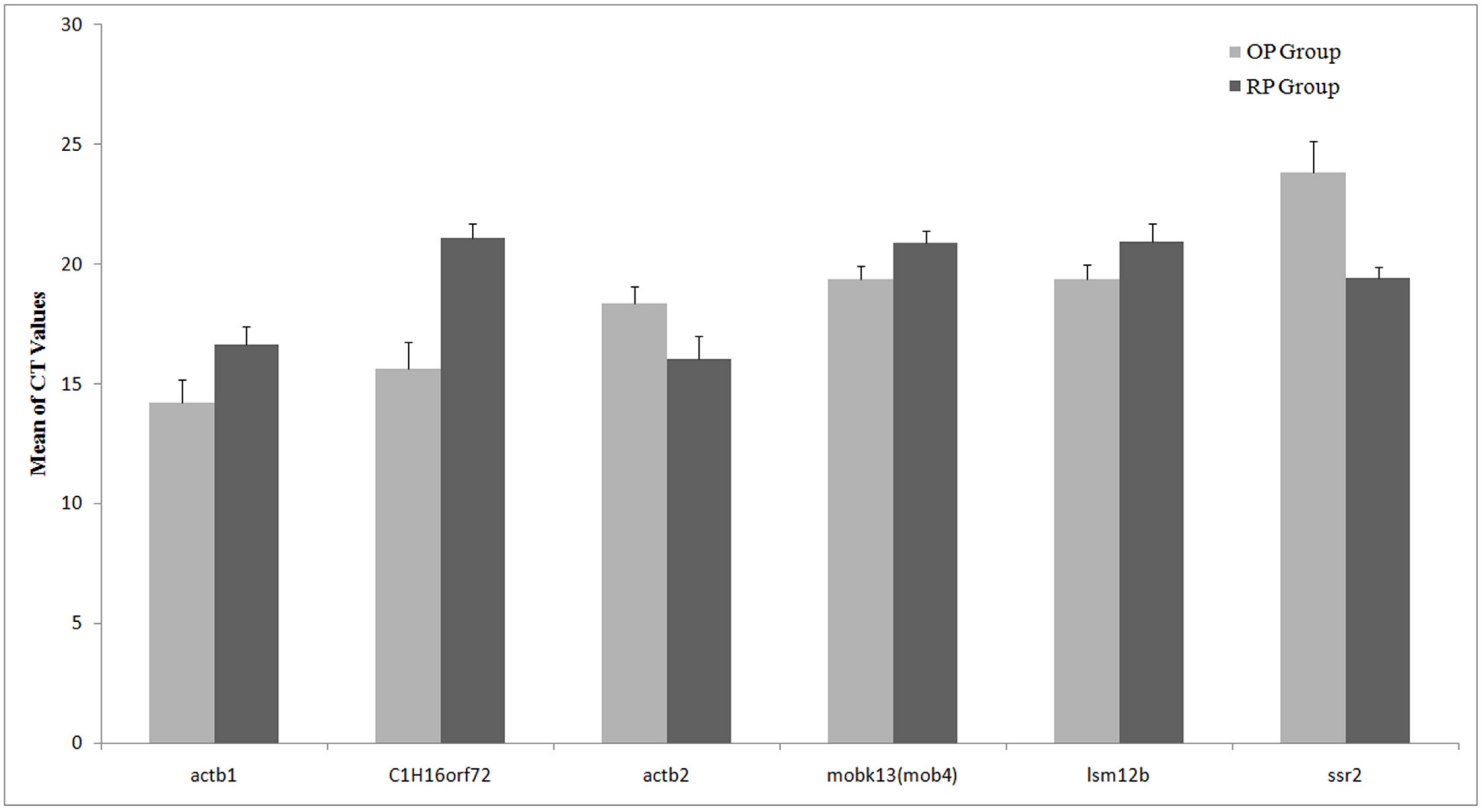
Mean CT and SD distribution of the six genes evaluated. Each set of charts shows the mean CT and SD for each gene evaluated. OP and RP groups are shown in light and dark grey, respectively. The short bars represent positive standard deviation values (SD).

To obtain reference genes appropriate for normalization in both the OP and RP groups, variations in the CT values between the two groups should be as small as possible. Fig 2 shows the mean and standard deviation (SD) of the original CT values of the six genes derived from qRT-PCR. We found that the genes with the smallest differences between OP and RP groups to be *lsm12b* and *mobk13*. The other 4 genes, namely *ssr2*, *C1H16orf72*, *actb1* and *actb2* showed larger differences, suggesting that *lsm12b* and *mobk13* may be more suitable as reference genes for zebrafish developmental studies.

Statistical methods were next applied to evaluate the expression stability of these genes. Keeping in mind that different approaches based on different principles may lead to disparate test results [27], we used four statistical algorithms, including delta-CT [33], geNorm [34], BestKeeper [35], together with NormFinder [36]. The most suitable reference genes were then determined according to a comprehensive evaluation of the results.

The delta-CT method ranks candidate reference genes based on their delta-CT values [33]. Genes with the lowest delta-CT values have the most stable expression. Table 2 shows the order of the expression stability of the tested genes. From the most stable to the least, they are *mobk13*, *lsm12b*, *actb2*, *actb1*, *C1H16orf72* and *ssr2* for the OP group, and *mobk13*, *C1H16orf72*, *ssr2*, *lsm12b*, *actb1* and *actb2* for the RP group. Regardless of group, *mobk13* and *lsm12b*were ranked before*actb1* and *actb2*, and *mobk13*was ranked first, which would indicate that it is the most stable gene during zebrafish early development.

**Table 2:** Stability ranking of the tested genes evaluated with four statistical methods.

**Ranking for OP Group**	**Delta-CT(Delta-CT Value)**	**geNorm (M Value)**	**BestKeeper (CV ± SD)**	**NormFinder (Stability Value)**
1	*mobk13*(0.82)	*lsm12b/actb2*(0.376)	*mobk13*(2.22±0.43)	*mobk13*(0.162)
2	*lsm12b*(0.86)	*lsm12b/actb2*(0.376)	*lsm12b*(2.26±0.44)	*lsm12b*(0.384)
3	*actb2*(0.89)	*mobk13*(0.490)	*actb2*(2.85±0.52)	*actb2*(0.410)
4	*actb1*(1.01)	*actb1*(0.711)	*actb1*(5.47±0.78)	*actb1*(0.692)
5	*C1H16orf72*(1.18)	*C1H16orf72*(0.817)	*C1H16orf72*(5.79±0.91)	*C1H16orf72*(1.027)
6	*ssr2*(1.51)	*ssr2*(1.047)	*ssr2*(4.12±0.98)	*ssr2*(1.413)
**Ranking for RP Group**	**Delta-CT (Delta-CT Value)**	**geNorm (M Value)**	**BestKeeper (CV ± SD)**	**NormFinder (Stability Value)**
1	*mobk13*(0.58)	*ssr2/lsm12b*(0.449)	*ssr2*(1.86±0.36)	*mobk13*(0.221)
2	*C1H16orf72*(0.64)	Ssr2/lsm*12b*(0.449)	*mobk13*(1.77±0.37)	*C1H16orf72*(0.379)
3	*ssr2*(0.67)	*mobk13*(0.470)	*C1H16orf72*(2.10±0.44)	*ssr2*(0.479)
4	*lsm12b*(0.69)	*C1H16orf72*(0.531)	*lsm12b*(2.35±0.49)	*lsm12b*(0.504)
5	*actb1*(0.80)	*actb1*(0.642)	*actb1*(3.55±0.59)	*actb1*(0.646)
6	*actb2*(0.81)	*actb2*(0.698)	*actb2*(4.91±0.79)	*actb2*(0.675)

The ranking of reference genes provided by the geNorm algorithm is based on the expression stability M value, which is derived from the average pairwise variation of a potential reference gene set with all other genes under investigation; the lower the M value, the higher the gene’s expression stability. With this method, not only one but two of the most stable genes were established, making it possible to further minimize potential systematic errors [34]. In addition, the optimal number of reference genes for normalization can also be determined with geNorm by analysis of pairwise variation (V_n/n + 1_) [46]. The ranking of the expression stabilities of the six genes by geNorm are shown in Table 2 and S1 Fig. For the OP group, the order was *lsm12b* = *actb2*, *mobk13*, *actb1*, *C1H16orf72* and *ssr2*, while for the RP group, it was *ssr2* = *lsm12b* followed by *mobk13*, *C1H16orf72*, *actb1* and *actb2*. Overall, the results showed that the most suitable reference gene is *lsm12b*, while *mobk13* may also be appropriate for normalization. However, as the ranking of *actb2* and *ssr2* fluctuated greatly in the OP and RP groups, they may not be ideal as applicable reference genes.

The program BestKeeper is a form of analysis similar to that of geNorm, but can also be used to find single stably expressed genes [35]. It evaluates each candidate in terms of its coefficient of correlation to an index consisting of the geometric mean of all candidates and calculates both the SD and CV of the original CT values [47]. The lowest calculated variations (CV ±SD) indicate candidate genes with the greatest stability [48]. In addition, it also evaluates the candidates by means of their coefficient of Pearson correlation to an index (BestKeeper) consisting of the geometric mean of all candidates and also with pair-wise correlation analysis [47]. According to the variability observed, the ranking from the most stably expressed to the least is as follows: *mobk13* > *lsm12b* > *actb2*> *actb1* > *C1H16orf72* > *ssr2* for the OP group, and *ssr2* > *mobk13* > *C1H16orf72* > *lsm12b* > *actb1* > *actb2* for the RP group. Evaluation by BestKeeper also showed that the genes more stable than *actb1* and *actb2* in both OP and RP groups were *mobk13* and *lsm12b*.

NormFinder, in contrast, is a model-based approach. It ranks the set of candidate genes according to their expression stability, which is calculated from the amount of linear scale expression transformed by delta-CT method [36]. The lowest stability value represents the lowest variation and the highest stable expression. The ranking of gene stability with this method is displayed in Table 2. In the OP group, the order of the stability values of candidates is as follows: *mobk13*, *lsm12b*, *actb2*, *actb1*, *C1H16orf72* and *ssr2*. This result was very similar with that of the other three methods. In the RP group, the ranking, namely *mobk13*, *C1H16orf72*, *ssr2*, *lsm12b*, *actb1* and *actb2* was the same as with delta-CT method, while it differed with the other two methods, especially in the ranking of the top four genes. In spite of this, *mobk13* and *lsm12b* were ranked before *actb1* and *actb2* in most cases, whether in the OP or RP group.

In summary, by evaluating the expression stability with four independent algorithms, we found that the top two stable genes in the OP group were *mobk13* and *lsm12b* except for *lsm12b* and *actb2* with geNorm. In the RP group, *mobk13* and *lsm12b* were always ahead of *actb1* and *actb2*, although the top two varied among *mobk13*, *lsm12b*, *ssr2* and *C1H16orf72* with different methods. That is to say, in most cases, *mobk13* and *lsm12b* were more stable than the commonly used reference genes *actb1* and *actb2*. Therefore, *mobk13* and *lsm12b* may be suitable as novel reference genes in studies of zebrafish early development.

Of note, the whole-mount in situ hybridization results (S2 and S3 Figs) also provided important evidence that *mobk13* and *lsm12b* may be suitable as reference genes in view of their broad expression during zebrafish early development.

### Normalization of three target genes with novel reference genes

**Fig 3:**
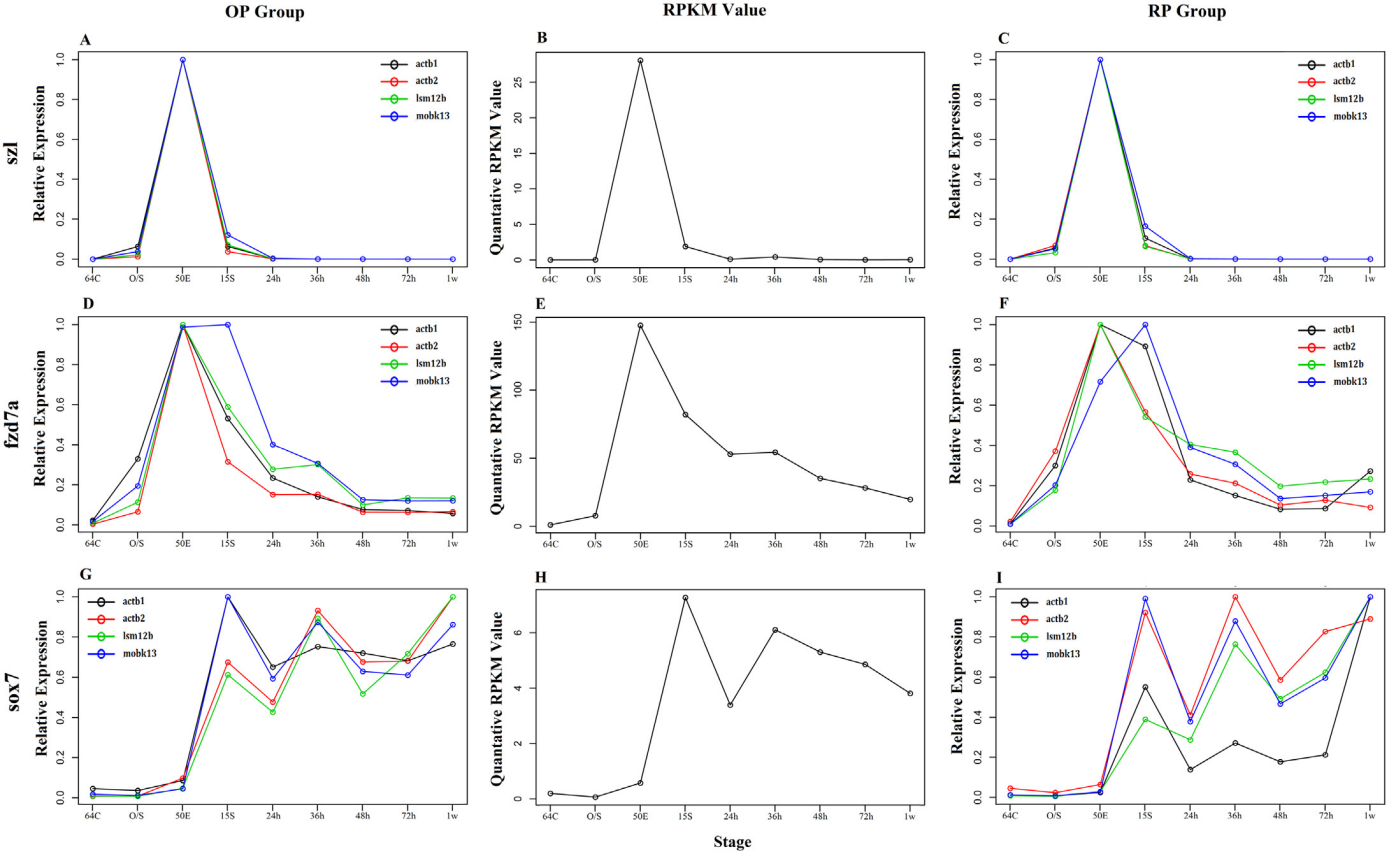
Normalization of three target genes with 4 reference genes. For the target genes, the real-time qRT-PCR derived relative expression levels (A, D and G for OP group. C, F and I for RP group), and the RPKM values (B, E and H) are shown. For the qRT-PCR results, the black circles and the line represent the relative expression levels normalized by *actb1*, the red circles and the line show those normalized by *actb2*, while the green and the blue ones represent those normalized by *lsm12b* and *mobk13*, respectively. The high consistency between RPKM values and the qRT-PCR derived expression levels suggests that all the four reference genes were appropriate for *szl* normalization, while *lsm12b*and *mobk13* are the best for *fzd7a* and *sox7* normalization, respectively.

To further test the suitability of *mobk13* and *lsm12b* as novel reference genes, they were used to normalize three well-studied target genes. As a comparison, *actb1* and *actb2* were also used. The target genes were *frizzled class receptor 7a* (*fzd7a*, involved in epiboly movement) [49], *sex determining region Y-box 7*(*sox7*, involved in vascular development) [50] and *sizzled* (*szl*, involved in dorsal-ventral patterning) [51]. Fig 3 shows normalization results of the target genes. The curves representing normalized data on the left and right helped to determine the most suitable reference genes when compared with the control (original RPKM values from RNA-Seq) in the middle. For *szl*, the curves of normalized CT values with four reference genes almost overlapped (Fig 3A and 3C), and their trends were similar to that of the RPKM (Fig 3B). In addition, there was good consistency between OP (Fig 3A) and RP groups (Fig 3C), suggesting that all four genes were appropriate for *szl* normalization. For *fzd7a*, the trends of the 4 normalized data were all consistent with the *fzd7a* RPKM curve (Fig 3D, 3E and 3F). Among them, the curves derived from *lsm12b* and *actb2* normalization were similar in shape to that of RPKM values, with that for *lsm12b*was almost the same as the RPKM curve of in both OP and RP groups. Therefore, we consider that *lsm12b* is the best reference gene for *fzd7a* normalization. Different from the above two, the normalization data of the *sox7* gene (Fig 3G, 3H and 3I) showed a relatively complicated result. The four curves were similar and consistent with the trend of the corresponding RPKM values of both OP and RP groups during the 64-cell stage to 50%-epiboly stage. After the 15-somite stage, curves of *mobk13* and *actb1* were most similar with that of RPKM in the OP group (Fig 3G and 3H). In the RP group, however, the curves representing *mobk13* and *actb2* normalization were most consistent with the RPKM curve, while the *actb1* curve was the least (Fig 3H and 3I). Thus, *mobk13* is the most suitable reference gene for *sox7* normalization during embryogenesis although it may need other genes for correction at the early larval stage.

Based on the above, *mobk13* and *lsm12b* are thought to be more stable and suitable as reference genes than both *actb1* and *actb2* for qRT-PCR normalization during zebrafish early development. However, a combined utilization of two or three internal reference genes is also recommended due to variation in reference gene expression. This is consistent with a previous report describing the necessity of using two to four references genes together for qRT-PCR normalization [30]. To our knowledge, this is the first study identifying *mobk13* and *lsm12b* as reference genes for zebrafish development studies. Our results will contribute to improvements in gene expression profile analyses, as well as functional studies of early development in zebrafish.

## Conclusions

Based on our previous data obtained with a whole-genome RNA-seq assay for zebrafish at embryonic and early larval stages, we focused on identifying and evaluating novel reference genes suitable for qRT-PCR normalization during zebrafish early development. Among 197 genes with a relatively stable expression, which were derived from our previous RNA-Seq dataset (29,291genes in total), two genes (*mobk13* and *lsm12b*) exhibited the greatest expression stability as determined using 4 independent statistical algorithms. Furthermore, the differences between the newly found genes and commonly used reference ones were also uncovered after normalization for target genes. *Mobk13* and *lsm12b* would be ideal as novel reference genes for qRT-PCR analysis during zebrafish development, contributing to more precise gene expression analysis and functional studies of early development in zebrafish.

## Supporting Information

S1 Fig Expression stability analysis of the tested genes by geNorm. A and B represent the OP and RP groups, respectively. The lower the M value calculated by geNorm, the higher the gene’s expression stability. In the OP group, the genes with the highest stable expression were *lsm12* and *actb2*, while the least stable was *ssr2*. In RP group, the genes with the most stable expression were *ssr2* and *lsm12*, and the least stable was *actb2*. Overall, the most stable gene was *lsm12*.
(TIF)

S2 Fig Expression patterns of *mobk13* in zebrafish embryos. *Mobk13* was strongly and widely expressed during early development; after 48hpf, the signals in somites decreased, butwere still strong in the head. All panels show a lateral view.
(TIF)

S3 Fig Expression patterns of *lsm12b* in zebrafish embryos. *Lsm12b* was expressed widely and strongly by the 15-somite stage; later, *lsm12b* signals declined in somites, while they appeared to be elevated in brain and spinal cord. All panels show a lateral view.
(TIF)

S1 Table List of primers used in this study. (XLS)

S2 Table The197 most stably expressed genes (RPKM_max/min_<2) screened from the RNA-Seq dataset (29,291 genes in total). (XLSX)

S3 Table 12 genes with a minimum RPKM value>40 selected from the 197 genes (RPKM_max/min_<2). (XLSX)

S4 Table Numbers of possible pseudogenes of the studied genes. (XLSX)

## Abbreviations

qRT-PCR: Quantitative real-time polymerase chain reaction
ERE: Expressed repetitive elements
RPKM: Reads per kilobase per million
CV: Coefficient of variation
BLAT: BLAST-Like alignment tool
CT: Cycle threshold
OP: oligo (dT)_15_ primer
RP: random primer
SD: standard deviation
