# Supplementary figures and images for: Identification of Novel Reference Genes Suitable for qRT-PCR Normalization with Respect to the Zebrafish Developmental Stage
Source: PLoS One. 2016 Feb 18;11(2):e0149277. doi: 10.1371/journal.pone.0149277 (PMC4758726; doi:10.1371/journal.pone.0149277)

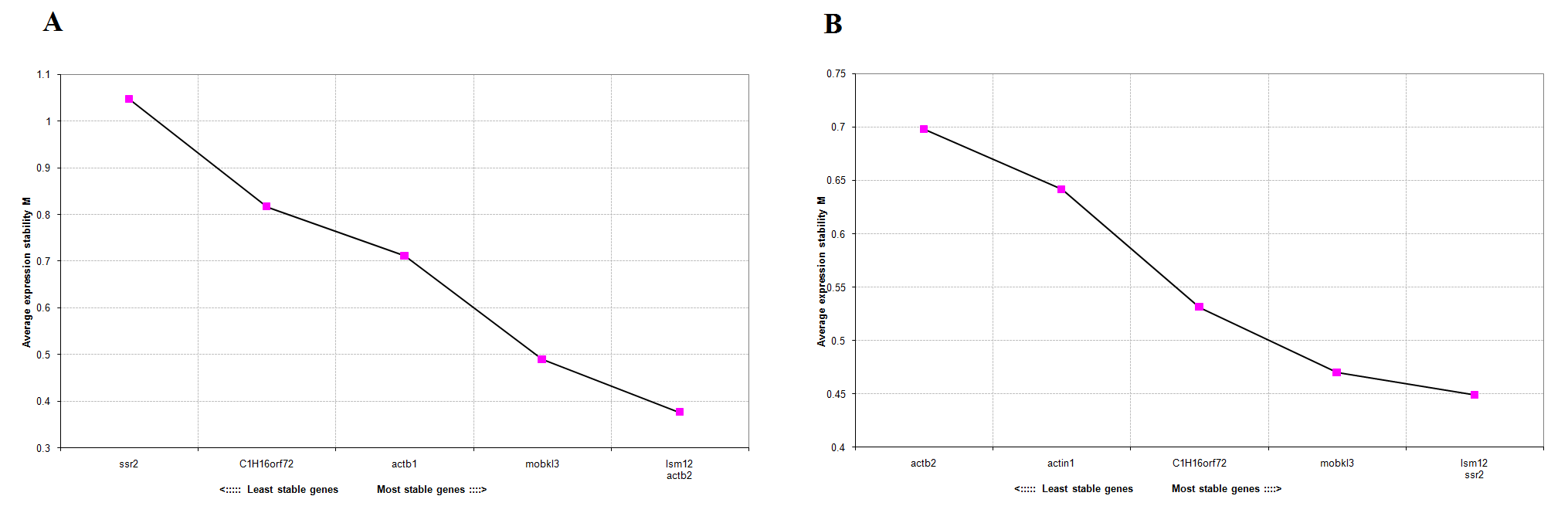

Supplement: S1 Fig — A and B represent the OP and RP groups, respectively. The lower the M value calculated by geNorm, the higher the gene’s expression stability. In the OP group, the genes with the highest stable expression were lsm12 and actb2, while the least stable was ssr2. In RP group, the genes with the most stable expression were ssr2 and lsm12, and the least stable was actb2. Overall, the most stable gene was lsm12. (TIF) [file pone.0149277.s001.tif]

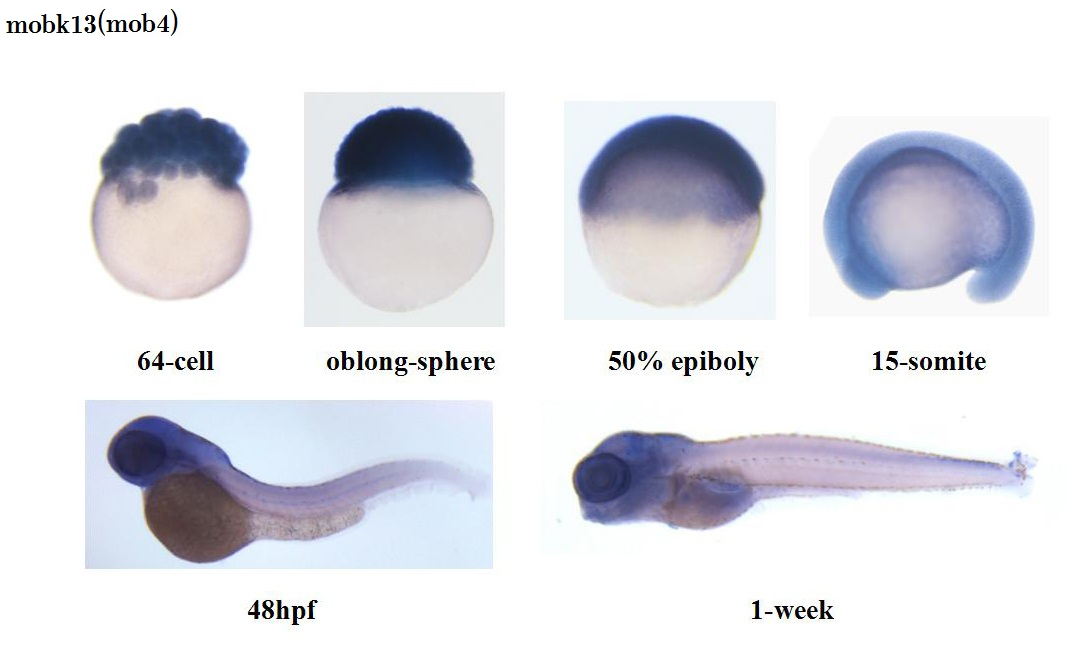

Supplement: S2 Fig — Mobk13 was strongly and widely expressed during early development; after 48hpf, the signals in somites decreased, butwere still strong in the head. All panels show a lateral view. (TIF) [file pone.0149277.s002.tif]

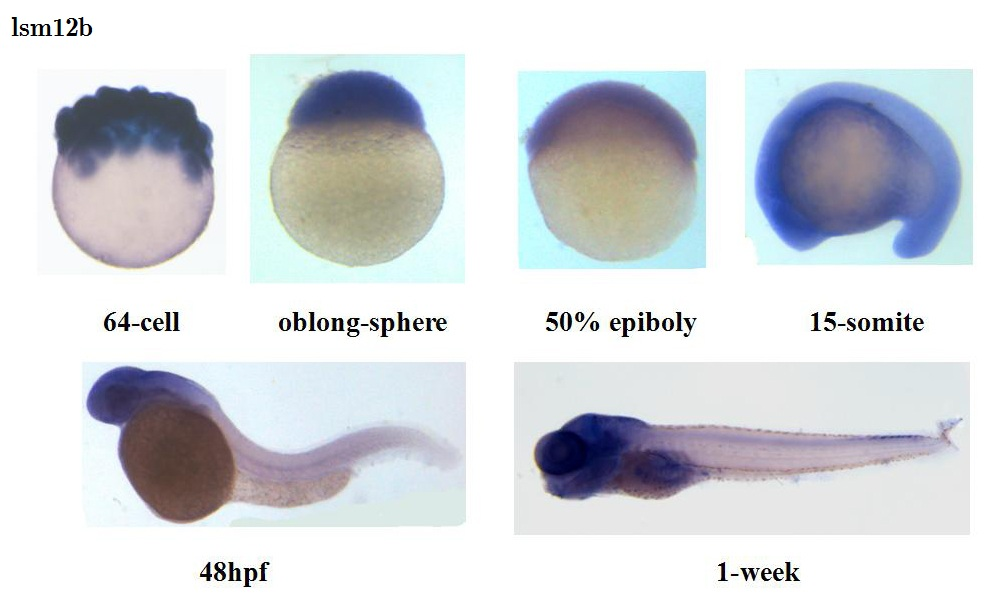

Supplement: S3 Fig — Lsm12b was expressed widely and strongly by the 15-somite stage; later, lsm12b signals declined in somites, while they appeared to be elevated in brain and spinal cord. All panels show a lateral view. (TIF) [file pone.0149277.s003.tif]
